# High transmissibility of norovirus among infants and school children during the 2016/17 season in Osaka, Japan

**DOI:** 10.2807/1560-7917.ES.2018.23.6.18-00029

**Published:** 2018-02-08

**Authors:** Naomi Sakon, Jun Komano, Heidi L. Tessmer, Ryosuke Omori

**Affiliations:** 1 Department of Microbiology, Osaka Institute of Public Health, Japan; 2 Department of Clinical Laboratory, Nagoya Medical Center, Japan; 3 Division of Bioinformatics, Research Center for Zoonosis Control, Hokkaido University, Sapporo, Hokkaido, Japan; 4 JST, PRESTO, 4-1-8 Honcho, Kawaguchi, Saitama, 332-0012, Japan

**Keywords:** norovirus, epidemiology, school outbreaks, modelling

## Abstract

The number of person-to-person transmitted norovirus cases (n = 4,712) in school children in Osaka, Japan, during 2016/17 was the largest since 2012/13. Norovirus outbreaks were reported by 101 schools including 53 nursery schools (1,927 cases), 18 kindergartens (1,086 cases) and 30 elementary schools (1,699 cases). The dominant genotype among outbreaks was GII.P16-GII.2 (57.4%; 58/101), followed by GII.P2-GII.2 (8.9%; 9/101) and GII.P7-GII.6 (5.9%; 6/101). GII.4 was not detected despite dominance in previous years.

In 2016/17, a large number of children attending schools or nurseries were affected by norovirus in the Osaka prefecture. During this time, the GII.2 genotype dominated in contrast to the GII.4 genotype, which had been majorly detected in previous years. To alert on these school/nursery outbreaks coinciding with a genotype shift before the next upcoming norovirus season, we hereby characterise the 2016/17 epidemic in the prefecture and compare it to previous seasons in the 2012 to 2016 period.

## Outbreak investigation

The Osaka prefecture, Japan, has a complete and continuous norovirus surveillance programme in the prefecture’s schools [1]. Norovirus outbreaks were investigated between April 2012 and March 2017 using the viral acute gastroenteritis (AGE) surveillance system established in the prefecture with the exception of the cities of Osaka (since inception), Hirakata (April 2014 onwards), and Sakai, Takatsuki, and Toyonaka (April 2013 onwards). The Osaka prefecture norovirus surveillance system has been described previously [1]. An AGE outbreak was defined as an instance in which more than 10 individuals developed gastrointestinal symptoms. All nursery schools, kindergartens, and junior high schools in Osaka prefecture are obligated to report such outbreaks to their regional public health centres, and public health officials are required to collect stool specimens from affected individuals for investigation. Laboratory diagnosis was performed at the Osaka Institute of Public Health using methods described previously [1]. From each norovirus outbreak, one to three randomly selected norovirus-positive specimens were sequenced. The norovirus NoroNet genotyping tool version 2.0 was used to identify the norovirus genotype (http://www.rivm.nl/en/Topics/N/NoroNet). The study protocol was approved by the ethics committee of the Osaka Institute of Public Health (number 0710–03–02).

## Estimation of incidence rate and effective reproduction number per school

To compare the norovirus transmission potential by sampling season we estimated the incidence rate and the effective reproduction number, *R*_e_. These values were estimated using the number of students per school where norovirus outbreaks were detected. The incidence rate was estimated by maximum likelihood estimation assuming a binomial sampling process. *R*_e_ was estimated by fitting a mathematical model, the individual-based susceptibles, exposed, infectious, recovered (SEIR) model [2], describing the norovirus transmission process in each school to the observed outbreak size per school. We assumed a constant latent period (24 hours) and infectious period (3.35 days) [2]. In estimating *R*_e_, approximate Bayesian computation was conducted using the following summary statistic (*S*) [3]:

*S* = ((Incidence rate from simulation)-(Incidence rate from data))/(Incidence rate from data).

For each school, 1,000,000 simulation runs were conducted to construct the posterior distributions for *R*_e_, and each simulation run used a different parameter value sampled from the non-informative prior for *R*_e_ (uniform distributions with a range of: 0–6).

## Comparisons across the 2012/13–2016/17 seasons

**Figure 1:**
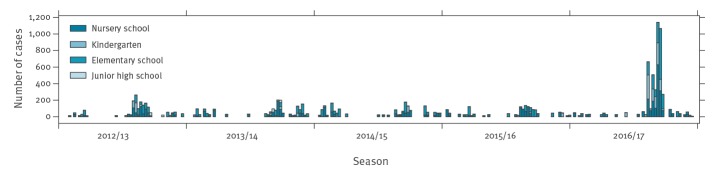
Number of norovirus infections among school children in Osaka prefecture, Japan, 2012/13–2016/17 seasons

**Figure 2:**
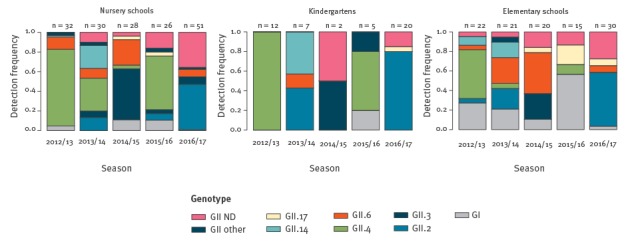
Time trend of frequencies of specific genotypes detected in norovirus infections among school children in Osaka prefecture, Japan, 2012/13–2016/17 seasons

During the 2016/17 season, the number of norovirus cases in school children was the largest since the 2012/13 season (Figure 1 and Table). The most frequently genotype detected was GII.P16-GII.2 (57.4%; 58/101), followed by GII.P2-GII.2 (8.9%; 9/101) and GII.P7-GII.6 (5.9%; 6/101). Due to lack of genotype data classified by RNA-dependent RNA polymerase (RdRp) for 2012/13–16/17, genotype data classified by capsid was used for all comparisons between 2012 and 2017. In contrast to 2016/17 when GII.2 dominated, over the 2012/13–2015/16 period, GII.4 was the major genotype affecting infants and school children (Figure 2). In the same period, GII.6 was also consistently identified but at low frequency in each season, especially in 2015/16 when it was barely detected. In addition, from 2014/15 onwards GII.17 also occurred but also at low frequency in each season.

**Table:** Summary of norovirus outbreaks among school children in Osaka prefecture, Japan, 2012/13–2016/17 seasons

Description of the schools and outbreaks		Season					
		2012/13	2013/14	2014/15	2015/16	2016/17	All
Nursery school	Number of outbreaks (total number of schools)	32 (NA^a^)	30 (NA^a^)	28 (NA^a^)	26 (NA^a^)	53 (NA^a^)	169 (NA^a^)
	Total reported cases	585	746	693	611	1,927	4,562
	Incidence (95% CI)	0.16 (0.15–0.17)	0.20 (0.19–0.21)	0.21 (0.19–0.22)	0.18 (0.17– 0.20)	0.31 (0.30–0.33)	0.24 (0.23–0.24)
	*R*_e_ (95% CI)	1.11 (0.54–3.34)	1.14 (0.60–2.48)	1.15 (0.60–2.43)	1.15 (0.61–2.40)	1.24 (0.69–2.62)	1.18 (0.61–2.71)
Kindergarten	Number of outbreaks (total number of schools)	12 (483)	7 (439)	2 (402)	5 (376)	18 (367)	44 (1,691)
	Total reported cases	273	131	93	181	1,086	1,764
	Incidence (95% CI)	0.19 (0.17–0.22)	0.23 (0.20–0.27)	0.12 (0.10–0.15)	0.22 (0.19–0.25)	0.30 (0.28–0.31)	0.24 (0.23–0.25)
	*R*_e_ (95% CI)	1.12 (0.60–3.00)	1.18 (0.59–2.76)	1.07 (0.63–2.25)	1.16 (0.64–2.24)	1.24 (0.67–2.44)	1.19, (0.63–2.62)
Elementary school	Number of outbreaks (total number of schools)	22 (589)	21 (545)	20 (497)	15 (495)	30 (485)	108 (2,611)
	Total reported cases	702	690	782	557	1,699	4,430
	Incidence (95% CI)	0.05 (0.05–0.06)	0.06 (0.05–0.06)	0.06 (0.06–0.06)	0.06 (0.06–0.07)	0.11 (0.10– 0.12)	0.07 (0.07–0,07)
	*R*_e_ (95% CI)	1.03 (0.56–2.48)	1.04 (0.57–2.12)	1.04 (0.59–2.12)	1.04 (0.59–2.29)	1.08 (0.65–1.77)	1.05 (0.59–2.12)
Junior high school	Number of outbreaks (total number of schools)	2 (306)	1 (286)	0 (266)	0 (263)	0 (259)	3 (1,380)
	Total reported cases	54	41	0	0	0	95
	Incidence (95% CI)	0.04 (0.03–0.05)	0.07 (0.05–0.09)	NA	NA	NA	0.05 (0.04–0.06)
	*R*_e_ (95% CI)	1.02 (0.56–2.08)	1.01 (0.64–1.72)	NA	NA	NA	1.02 (0.58–1.96)
All	Number of outbreaks (total number of schools)	68 (NA^a^)	59 (NA^a^)	50 (NA^a^)	46 (NA^a^)	101 (NA^a^)	324 (NA^a^)
	Total reported cases	1,614	1,608	1,568	1,349	4,712	10,851
	Incidence (95% CI)	0.08 (0.08–0.09)	0.10 (0.09–0.10)	0.09 (0.09–0.09)	0.11 (0.11–0.12)	0.19 (0.18–0.19)	0.12 (0.12–0.12)
	*R*_e_ (95% CI)	1.07 (0.55–3.02)	1.10 (0.59–2.44)	1.08 (0.59–2.34)	1.10 (0.61–2.34)	1.16 (0.67–2.43)	1.11 (0.61–2.53)

CI: confidence interval; NA: not applicable;* R*_e_: effective reproduction number at the initial phase of an outbreak.

^a^ The total number of nursery schools is not available.

ND: non-determined genotype.

The numbers above the chart bars (e.g. n = 32, n = 30 etc.) are the numbers of norovirus outbreaks.

Comparing the number of cases in the 2016/17 season to the average number of cases per season from 2012/13–2015/16, the number of cases was higher at 193% (1,927 vs 659), 539% (1,086 vs 170), and 149% (1,699 vs 683) in nursery schools, kindergartens, and elementary schools, respectively (Table). Comparing the 2016/17 season to each of the seasons from 2012/13–2015/16, the number of cases was higher with a range of 158.3–229.4%, 297.8–1,067.7%, 117.2–205.0%, in nursery schools, kindergartens, and elementary schools, respectively.

Comparing the proportion of schools reporting outbreaks in the 2016/17 season to the average proportion per season from 2012/13–2015/16, the proportion was higher at 221% (18/367 vs 26/1,700) and 69% (30/485 vs 78/2,126) in kindergartens and elementary schools, respectively (Table). The proportion for nursery schools was not calculated as the total number of schools in the prefecture was not available. Comparing the 2016/17 season to each of the seasons from 2012/13–2015/16, the proportions were higher with a range of 97.4–885.8% and 53.7–104.1%, respectively (Table).

During the 2016/17 season, a significantly higher incidence rate and effective reproduction number, *R*_e_, were observed in nursery schools and elementary schools compared with other seasons (Table) (Wilcoxon rank sum test with Holm-Bonferroni correction, p < 0.05). Conversely, no significant differences were found among the norovirus seasons between 2012/13 and 2015/16. Similar trends for the school-specific number of cases, incidence rate, and *R*_e_ over multiple seasons were observed among kindergartens, and the differences between all pairs of seasons were not significant. In junior high schools both incidence and *R*_e_ tended to decrease over seasons, and no outbreaks were observed from the 2014/15 to 2016/17 seasons.

Over all seasons, nursery schools showed the largest number of outbreaks, reported cases, incidence rate, and *R*_e_, followed by elementary schools, kindergartens, and junior high schools (Table).

## Discussion

Previous population-based surveys estimated the reproduction number of norovirus to be in the range of 0.14 to 17.98 [4-9]. Our estimated reproduction number range, 0.54 to 3.34 in school children, was similar to those previously found in England and Wales (0.89 for people over 5 years of age) [4] and Japan (0.14 to 4.15 for all age groups) [5].

Various norovirus genotypes circulate among infants and children at schools and the dominant genotypes change almost every year, as shown in Figure 2 [1]. The norovirus genotype most frequently detected in 2016/17 was GII.P16-GII.2 (55.4%), followed by GII.P2-GII.2 (8.9%) and GII.P7-GII.6 (5.9%), which is similar to other countries in 2016/17 [10,11]. Comparing available genotype data classified by capsid between 2012 and 2017, indicated that GII.2 sampled during the 2016/17 season had significantly higher transmissibility than GII.2 sampled during the 2012/13–2015/16 seasons both in terms of incidence rate and *R*_e_ (Wilcoxon rank sum test with Holm-Bonferroni correction, p < 0.05). A significant increase in transmissibility was also observed in GII.6. In contrast, GII.4 was not detected in school children during the 2016/17 season despite having been present throughout the 2012/13 to 2015/16 seasons with overall similar incidence rates and *R*_e_s. A statistically significant difference was not observed in comparisons of GII.4 incidence rates and *R*_e_s across pairs of seasons from 2012/13 to 2015/16, except between 2012/13 and 2015/16 (Wilcoxon rank sum test with Holm-Bonferroni correction, p < 0.05). It is notable that although detected in the Osaka Prefecture surveillance programme in adults (19.1% of cases) (data not shown), norovirus GII.17 was detected infrequently in infants and children in the 2016/17 season (2.9% of cases).

Norovirus transmissibility in school children during 2016/17, when the dominant genotype was GII.2, was significantly higher than the transmissibility during 2012/13–15/16, when the dominant genotype was GII.4. During the 2016/17 season, a significant increase in transmissibility was also observed for GII.6. It is plausible that the high transmissibility of both GII.2 and GII.6 is correlated with a genotype shift of norovirus in infants and children. Two primary factors could contribute to make this happen: a naïve population and viral mutations. Firstly, among all kindergartens, nursery schools, and elementary schools, GII.2 and GII.6 had been rare in the four seasons preceding the 2016/17 season, so it is likely that a large population naïve to GII.2 and GII.6 had accumulated due to student turnover. Secondly, norovirus mutant strains of GII.2 and GII.6, with increased epidemic potential and/or ability to overcome infants and children herd immunity may have been selected. While genetic analysis of GII.6 strains remains to be performed in depth, a greater number of mutation were found in GII.2 during the 2016/17 season compared with previous seasons [11,12,13]. Although these mutations were positioned at non-B-cell epitopes, such mutations could possibly yield novel properties leading to epidemics [13,14]. These two factors, in combination, may have contributed to the infants and children of the 2016/17 season being predisposed to infection by norovirus GII.2 and GII.6.

While the above two factors may have contributed to the increased transmissibility of GII.6, other factors may explain why the number of outbreaks with this genotype remained small despite the increasing effective reproduction number, such as (hypothetically) epidemiological interference (possibly due to some cross immunity) between GII.6 and GII.2. The latter conjecture, however, remains to be investigated.

## Conclusion

The number of cases, incidence rate, and *R*_e_ in school children in Osaka, Japan, during 2016/17 were significantly higher than 2012/13–2015/16. During the 2016/17 season, compared with the 2012/13–2015/16 seasons, the number of cases increased by 193%, 539%, and 149% in nursery schools, kindergartens, and elementary schools, respectively. The proportion of kindergartens and elementary schools reporting outbreaks also increased by 221% and 69% respectively. Despite being rare in the 2012/13–2015/16 seasons, the dominant genotype in 2016/17 was GII.2 (66.3%; 67/101). In addition to the significant increase in incidence rate and *R*_e_ for GII.2, a significant increase was also observed for GII.6.
